# Ferroptosis in osteoarthritis: metabolic reprogramming, immunometabolic crosstalk, and targeted intervention strategies

**DOI:** 10.3389/fimmu.2025.1604652

**Published:** 2025-06-06

**Authors:** Shenglin Xia, Li Li, Zhexiong Shi, Nianyi Sun, Yu He

**Affiliations:** ^1^ Department of Rehabilitation, Shengjing Hospital of China Medical University, Shenyang, China; ^2^ Department of Rehabilitation, Shanghai Fourth People’s Hospital, School of Medicine, Tongji University, Shanghai, China; ^3^ School of Medicine, Tongji University, Shanghai, China

**Keywords:** osteoarthritis, ferroptosis, metabolic reprogramming, immunometabolic crosstalk, iron homeostasis, lipid peroxidation, inflammatory microenvironment

## Abstract

Osteoarthritis is a common degenerative joint disease characterized by progressive cartilage loss, bone remodeling, and chronic joint inflammation, yet its underlying mechanisms remain incompletely understood. Disrupted iron metabolism, particularly iron accumulation in joint tissues, contributes to oxidative damage and inflammation, suggesting a potential link to disease progression. This review focuses on ferroptosis, a regulated form of cell death driven by iron-dependent lipid peroxidation, as a key pathological mechanism in osteoarthritis. We summarize current evidence showing how impaired iron homeostasis, weakened antioxidant defenses, and metabolic alterations make chondrocytes and other joint cells vulnerable to ferroptotic injury. We further describe how inflammatory and metabolic signals interact to amplify ferroptosis, creating a self-reinforcing cycle of tissue damage. Finally, we explore emerging strategies to target ferroptosis, including iron chelation, antioxidant therapy, inhibition of lipid peroxidation, and gene or cell-based interventions. By integrating these findings, this review offers new insights into the role of ferroptosis in joint degeneration and highlights its potential as a therapeutic target in osteoarthritis.

## 1 Introduction

Osteoarthritis (OA) is among the most prevalent degenerative joint diseases globally, affecting more than 500 million people worldwide and ranking as the leading cause of disability among adults aged 50 years and older (1, 2). Primarily characterized by progressive degradation of articular cartilage, subchondral bone sclerosis, synovial inflammation, and weakened periarticular muscle strength (1), OA poses a substantial socioeconomic burden, with direct and indirect healthcare costs estimated at approximately 1–2.5% of the gross domestic product (GDP) in developed countries (2). As the global population rapidly ages, the incidence and prevalence of OA continue to rise sharply, significantly impairing patients’ quality of life and increasing pressure on healthcare systems worldwide (3). Traditionally, OA has been regarded as a purely mechanical “wear-and-tear” condition. However, accumulating evidence over recent years has revealed that the onset and progression of OA are also profoundly influenced by immune-inflammatory responses, metabolic reprogramming, oxidative stress, and multiple forms of programmed cell death (4).

Ferroptosis, first reported in 2012, is a novel form of cell death distinct from classical apoptosis, pyroptosis, and necrosis (5). Its main feature is uncontrolled lipid peroxidation triggered by disrupted iron homeostasis, ultimately causing irreversible damage to cellular structure and function (6). Given that iron dysregulation in articular cartilage, synovium, and subchondral bone may exacerbate inflammation, tissue damage, and degenerative processes, ferroptosis has been proposed as a key contributor to OA pathogenesis. Studies have shown that blocking or slowing ferroptosis may ameliorate OA-related cartilage damage and inflammatory responses, offering new possibilities for early diagnosis and intervention in OA (7, 8).

Due to the possible critical role of ferroptosis in OA, there has been a surge of interest in elucidating its molecular mechanisms and exploring potential therapeutic strategies. Nonetheless, many uncertainties and challenges remain, particularly regarding specific ferroptosis pathways, immunometabolic regulation, and clinical translational applications. Therefore, this review will focus on the following aspects: (a) A basic overview of OA and ferroptosis, highlighting the pathological features of OA as well as the discovery, definition, and core regulatory mechanisms of ferroptosis. We also summarize current evidence on ferroptosis involvement in OA progression. (b) The role of metabolic reprogramming in ferroptosis, analyzing how lipid, amino acid, iron, and glucose metabolism are reshaped or disrupted in OA, thereby influencing the occurrence and progression of ferroptosis. (c) Immunometabolic crosstalk, discussing the complex interactions among local immune signaling, metabolic pathways, and ferroptosis within the inflammatory microenvironment of osteoarthritic joints. (d) Ferroptosis-targeted therapeutic strategies and future perspectives, summarizing current ferroptosis-directed interventions that encompass pharmacological agents, gene therapy, and cell-based treatments, and examining both the challenges and opportunities of their clinical translation.

By systematically reviewing and synthesizing existing literature, this article aims to provide insights for further investigation into ferroptosis mechanisms in OA and to broaden potential therapeutic targets and approaches for clinical management of ferroptosis-related pathological processes. We hope these perspectives will inspire valuable directions for future basic and translational research in the field of OA diagnosis and treatment.

## 2 Overview of osteoarthritis and ferroptosis

### 2.1 Pathogenic mechanisms of osteoarthritis

OA is a common degenerative joint disease marked by gradual cartilage degradation, destruction of subchondral bone structure, synovial inflammation, and clinical symptoms such as joint pain. As the global population continues to age, the incidence and disability rate of OA steadily rise, creating significant economic and caregiving burdens on society and families. Although OA pathogenesis is exceedingly complex, recent studies underscore the multifactorial nature of the disease, involving mechanical overload, inflammatory responses, metabolic abnormalities, and cell death (9).

Cartilage deterioration and subchondral bone remodeling are hallmarks of OA. Articular cartilage is primarily composed of chondrocytes and an extracellular matrix (ECM), which consists mainly of type II collagen and proteoglycans (10). In OA, chondrocyte activity declines, leading to reduced synthetic capacity and increased secretion of matrix-degrading enzymes such as matrix metalloproteinase 13 (MMP-13) and a disintegrin and metalloproteinase with thrombospondin motifs 5 (ADAMTS-5). These enzymes degrade the ECM and cause a gradual loss of cartilage elasticity and shock-absorbing capacity (11). Meanwhile, under excessive mechanical stress and inflammatory stimuli, the subchondral bone experiences sclerosis, trabecular thickening, and osteophyte formation. The interactions between cartilage and subchondral bone further accelerate OA progression (12).

Synovial inflammation and immune dysregulation are also characteristic of OA. Although the degree of inflammation in OA is generally lower than in rheumatoid arthritis, synovial tissue can still exhibit marked hyperplasia and inflammatory cell infiltration (1). Macrophages, T lymphocytes, and B lymphocytes in the synovial fluid secrete proinflammatory cytokines such as interleukin-1β (IL-1β), tumor necrosis factor α (TNF-α), and interleukin-6 (IL-6). These cytokines not only accelerate degradation of the cartilage matrix but also amplify the local inflammatory response via autocrine or paracrine signaling (13). Research suggests that early intervention targeting OA-related synovitis may help delay disease progression and reduce pain (14).

**Figure 1:**
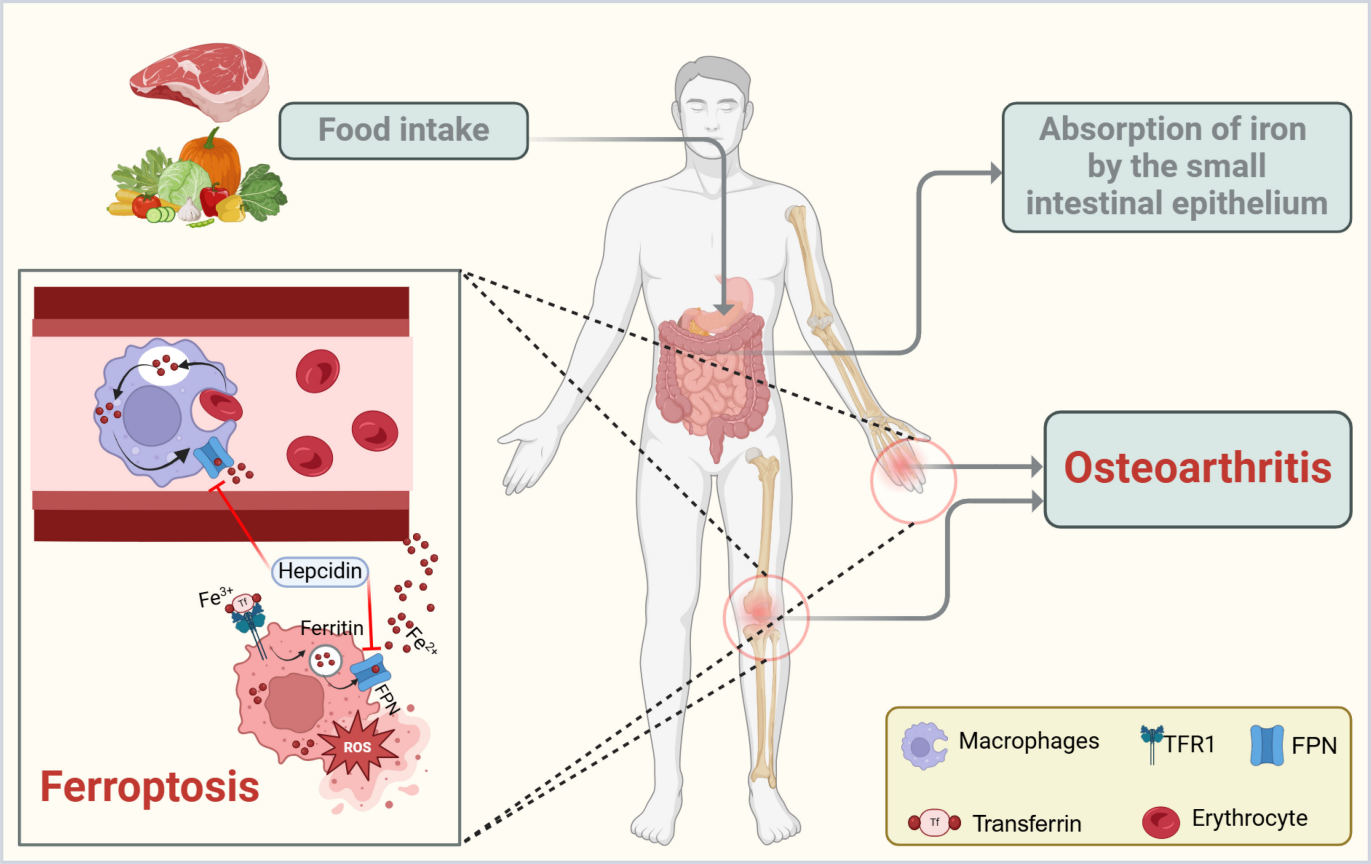
Schematic representation of iron handling and ferroptosis in OA. Dietary iron is absorbed through the small intestinal epithelium and circulated in the bloodstream, primarily bound to transferrin (Tf). Macrophages help recycle iron from erythrocytes and release it via ferroportin (FPN), a process that can be inhibited by the hormone hepcidin. Dysregulation of this pathway leads to intracellular iron overload, which drives ferroptosis by promoting lipid peroxidation and oxidative stress. In OA, excessive iron and inflammatory cytokines within the joint microenvironment further exacerbate ferroptosis, contributing to cartilage breakdown and disease progression. The figure was created with BioRender (https://biorender.com/).

Accompanying inflammation and mechanical damage, oxidative stress and multiple forms of cell death are deeply involved in the molecular mechanisms driving OA progression (15). Elevated levels of reactive oxygen species (ROS) in osteoarthritic joints can directly damage proteins, lipids, and nucleic acids, triggering several cell death pathways, including apoptosis, pyroptosis, and autophagy-related cell death (16). In recent years, it has become increasingly recognized that ferroptosis may hold a pivotal position in OA, as its distinctive iron-dependent lipid peroxidation mechanism offers a unique explanation for the link between local inflammation and iron overload (**Figure 1**).

### 2.2 Fundamental concepts and molecular mechanisms of ferroptosis

Ferroptosis was first described by Dixon in 2012 as an iron-dependent form of cell death featuring uncontrolled lipid peroxidation. It differs significantly from traditional apoptosis, pyroptosis, and necrosis (5).

#### 2.2.1 Lipid peroxidation and Fenton reactions

Lipid peroxidation is the core molecular event in ferroptosis (17). Phospholipids (PLs) rich in polyunsaturated fatty acids (PUFAs) are critical components of cell membranes. When PUFAs are excessively attacked by ROS, lipid hydroperoxides (LOOH) are generated (18). During Fenton reactions, ferrous iron (Fe2+) reacts with hydrogen peroxide (H2O2) and other peroxides to produce hydroxyl radicals (·OH), further amplifying the chain reaction of lipid peroxidation. Ultimately, the accumulation of lipid peroxidation byproducts severely compromises membrane integrity, causing irreversible cell death (19).

#### 2.2.2 Glutathione Peroxidase 4 (GPX4) and its role in ferroptosis

GPX4 is a selenoprotein that plays a central role in suppressing ferroptosis by catalyzing the reduction of membrane-bound phospholipid hydroperoxides (PL-OOH) into their corresponding non-toxic alcohols (PL-OH), thereby preserving lipid bilayer integrity under oxidative stress (17). This detoxifying reaction depends on reduced glutathione (GSH) as an electron donor, linking GPX4 activity directly to the availability of intracellular cysteine, which is imported via System Xc−. This transporter consists of the subunits SLC7A11 and SLC3A2 and exchanges extracellular cystine for intracellular glutamate (20). Depletion of GSH or inactivation of GPX4 leads to the accumulation of lipid peroxides and ferroptotic cell death (21, 22).

Beyond its classical enzymatic role, GPX4 is subject to multifaceted regulation. Structurally, it is the only GPX isoform with a monomeric conformation and a unique catalytic tetrad including Sec, Gln81, Trp136, and Asn137 that enables it to reduce complex hydroperoxides *in situ* even within membranes (23). This structural distinction underpins its irreplaceable role in ferroptosis control and embryonic development.

Transcriptionally, GPX4 expression is promoted by Nrf2 and ATF4 in response to oxidative or endoplasmic reticulum stress. Nrf2-driven antioxidant programs including the HO-1/Nrf2/GPX4 axis have demonstrated protective effects in various diseases such as stroke and renal injury (24). Vitamin-derived signals also influence GPX4. For instance, vitamin B6 supports GSH synthesis and restores GPX4 expression under oxidative stress, while vitamins E and K can act synergistically by directly blocking lipid peroxidation propagation (25).

At the post-transcriptional level, GPX4 is regulated by non-coding RNAs. MicroRNAs such as miR-214-3p and miR-15a-3p bind to the 3’ untranslated region of GPX4 mRNA and suppress its translation, sensitizing cells to ferroptosis. Conversely, long non-coding RNAs and circular RNAs act as molecular sponges that preserve GPX4 expression by sequestering these microRNAs. Such RNA-based networks have been implicated in ferroptosis regulation across diverse diseases including cancer, cardiovascular disease, and brain injury (24).

Pharmacologically, GPX4 remains a compelling yet challenging target. Recent studies have explored small-molecule inducers, selenium-based compounds, and targeted nanodelivery approaches to restore GPX4 function or mimic its antioxidant capacity in ferroptosis-prone diseases, with therapeutic implications for OA and beyond (26). These approaches are currently under investigation in neurodegeneration and cancer for their ability to modulate ferroptotic sensitivity.

In summary, GPX4 acts not only as a terminal antioxidant enzyme but also as a hub that integrates transcriptional, post-transcriptional, and nutritional cues. Its pivotal position in ferroptosis regulation highlights its importance as a therapeutic target for OA and other ferroptosis-driven diseases.

#### 2.2.3 Key regulatory networks

The tumor suppressor p53 can transcriptionally repress SLC7A11 expression, diminishing cystine uptake, curtailing GSH synthesis, and thereby promoting ferroptosis (27). Correspondingly, in OA, p53 signaling may be activated by oxidative stress, DNA damage, or other factors, contributing to cartilage injury (28).

Nuclear factor erythroid 2-related factor 2 (Nrf2) is a key transcription factor regulating redox homeostasis; it upregulates heme oxygenase-1 (HO-1) and other antioxidant and detoxification genes (29). In certain contexts, HO-1 (heme oxygenase-1) overexpression can also facilitate iron release; however, this process plays a pivotal role in iron overload, particularly in intestinal epithelial cells and macrophages responsible for iron recycling. HO-1 catalyzes the degradation of heme into biliverdin, carbon monoxide (CO), and Fe²^+^ by reducing the heme-associated ferric iron (Fe³^+^) to Fe²^+^ (30). The liberated Fe²^+^ must then be tightly regulated, either stored within ferritin or exported by FPN, to avoid intracellular accumulation and subsequent ferroptosis (31). In macrophages, especially those recycling senescent red blood cells in the spleen and liver, dysregulation of HO-1 or impaired iron export can contribute to local iron deposition and oxidative stress (32–34). Therefore, while HO-1 exerts cytoprotective effects under stress, its upregulation may paradoxically exacerbate iron-dependent cell injury when downstream iron-handling pathways are overwhelmed or dysfunctional. Studies indicate that activating the Nrf2 pathway significantly helps suppress lipid peroxidation and maintain iron homeostasis, making it a potential therapeutic target (35, 36).

Hepcidin can induce the internalization and degradation of FPN, thereby reducing cellular iron export and elevating intracellular iron levels (37). In OA, iron accumulation in the joint microenvironment is associated with oxidative stress and chondrocyte injury, potentially contributing to ferroptosis-related cartilage degeneration (38). IL-6, a key proinflammatory cytokine, is upregulated in multiple OA-related joint compartments, including synovial tissue, cartilage, and synovial fluid, where it promotes matrix degradation and inflammation (39). Although IL-6 is known to induce hepatic hepcidin expression via the JAK2/STAT3 pathway under systemic inflammatory conditions, current evidence does not support a significant increase in hepcidin expression within chondrocytes or synovial cells in OA (40). In fact, comparative analyses of synovial tissues show that hepcidin levels in OA are not significantly different from healthy controls, while FPN expression is upregulated, suggesting a compensatory mechanism to promote iron efflux (41). These findings indicate that disrupted iron homeostasis in OA may be more closely related to impaired iron export or excessive uptake, rather than local hepcidin overproduction.

In addition to these canonical signaling pathways, recent single-cell transcriptomic studies have revealed marked heterogeneity in ferroptosis susceptibility among chondrocyte subpopulations in OA cartilage. Lv et al. analyzed approximately 17,000 single cells from human OA cartilage and identified a ferroptosis-prone chondrocyte cluster (C1-3-4) with distinct expression of ferroptosis-related genes. This cluster showed elevated levels of inducers such as NCOA4 and Tf, along with reduced expression of the protective gene FTH1. Functional enrichment indicated strong activation of iron transport and lipid peroxidation pathways, but weak engagement of glutathione metabolism, suggesting selective vulnerability to iron-driven oxidative stress. This subcluster was predominantly located in severely degenerated cartilage regions and interacted frequently with regenerative clusters (C1-2, C1-4, C2), implying that ferroptotic cells may impair cartilage repair through paracrine effects. In addition, TRPV1, a potential protective regulator, was specifically downregulated in these cells. Activation of TRPV1 restored GPX4 levels, reduced lipid peroxidation, and improved cartilage integrity in both IL-1β–treated chondrocytes and OA mouse models (42).

These findings highlight the importance of considering cellular heterogeneity in ferroptosis regulation and suggest that targeting ferroptosis in a subpopulation-specific manner may enhance therapeutic precision in OA.

#### 2.2.4 FSP1 and other emerging regulatory factors

Alongside the critical role played by the GPX4 system, the ferroptosis suppressor protein 1 (FSP1)-coenzyme Q10 (CoQ10) pathway independently regulates ferroptosis (43). FSP1 (also known as AIFM2) is an NADH- and FAD-dependent oxidoreductase primarily located in the plasma membrane and lipid droplets (44). Mechanistically, FSP1 reduces CoQ and vitamin K, generating radical-trapping antioxidants (RTAs) that effectively terminate lipid peroxidation chain reactions at the plasma membrane, thereby guarding against ferroptosis. Unlike the GPX4 system, which relies on GSH to detoxify lipid hydroperoxides, FSP1 provides an alternative defensive route by generating RTAs through the reduction of CoQ10 and vitamin K. Notably, the N-terminal region of FSP1 undergoes irreversible myristoylation with a 14-carbon saturated fatty acid, which facilitates its anchorage to the plasma membrane. This post-translational modification is critical for its ferroptosis-suppressive function (43, 45). Loss or dysfunction of FSP1 sensitizes cells to ferroptosis (46). Moreover, FSP1 is important for CoQ10 synthesis and regeneration, helping cells withstand oxidative damage arising from excessive lipid peroxidation (47). By complementing the GPX4 system, the FSP1-CoQ10 axis bolsters the cell’s defense arsenal against oxidative stress and broadens our understanding of ferroptosis regulation and related therapeutic targets.

Additionally, recent findings have identified GTP cyclohydrolase-1 (GCH1), dihydroorotate dehydrogenase (DHODH), and several other factors as ferroptosis regulators, further expanding the ferroptosis network and offering new directions for elucidating ferroptosis in OA (48–50).

### 2.3 Overview of ferroptosis-associated changes in OA tissues

Recent studies have established a strong association between ferroptosis and the pathological features of OA, including cartilage degeneration, subchondral bone remodeling, and synovial inflammation. In OA cartilage tissues and IL-1β–stimulated chondrocyte models, iron accumulation, elevated levels of lipid peroxidation byproducts such as malondialdehyde (MDA) and 4-hydroxynonenal (4-HNE), and downregulation of the antioxidant enzyme GPX4 have been widely reported (51–53). Ferroptosis-related proteins such as ACSL4, SLC7A11, and FTH1 are also differentially expressed. Intervention with ferroptosis inhibitors such as ferrostatin-1 and deferoxamine has been shown to restore chondrocyte viability, reduce MMP13 and ADAMTS5 expression, and partially rescue extracellular matrix loss, suggesting that inhibiting ferroptosis may mitigate OA cartilage injury (54, 55).

Ferroptosis-related alterations are not limited to cartilage. In subchondral bone, chronic mechanical stress leads to microfracture, osteosclerosis, and angiogenesis, often accompanied by excessive iron deposition and oxidative stress. This creates a pro-ferroptotic microenvironment for osteoblasts and osteocytes, contributing to abnormal bone remodeling (56). In the synovium, fibroblast-like synoviocytes and infiltrating immune cells secrete proinflammatory cytokines such as IL-1β, IL-6, and TNF-α. These factors disrupt iron homeostasis by promoting hepcidin expression and inhibiting ferroportin-mediated iron export, thereby increasing intracellular free iron levels. Recent findings have shown that OA synovial exosomes carrying miR-19b-3p enhance ferroptosis in chondrocytes by targeting SLC7A1 (57), highlighting the pathological crosstalk between synovium and cartilage.

Ferroptosis also appears to correlate with OA clinical phenotypes. Elevated intra-articular iron levels and altered expression of ferroptosis-related molecules such as ACSL4 and GPX4 have been associated with the severity of joint pain, cartilage damage, and functional decline (58–60). These changes are detectable even in the early stages, suggesting that ferroptosis may serve as an initiating molecular event. Moreover, ferroptotic stress may not only contribute to tissue damage but also release proinflammatory mediators and damage-associated molecular patterns (DAMPs), reinforcing inflammation and accelerating tissue degeneration.

In summary, ferroptosis is a shared pathological mechanism across multiple joint tissues in OA. It links cartilage injury, bone remodeling, and synovial inflammation through a network of iron overload, oxidative stress, and metabolic disturbance. These findings set the stage for the mechanistic exploration in Sections 3 and 4, where we discuss how metabolic reprogramming and immune interactions converge to regulate ferroptosis in OA.

## 3 Metabolic reprogramming related to ferroptosis in OA

Having established the significance of ferroptosis in OA pathology, we now turn our attention to how metabolic reprogramming involving lipids, amino acids, iron, and glucose underpins ferroptotic susceptibility.

Metabolic reprogramming has been a focal point in the study of numerous diseases in recent years, with well-established theories, particularly in oncology. In OA and other chronic degenerative disorders, cells and tissues also undergo shifts in energy metabolism and biosynthetic pathways to adapt to pathological microenvironments characterized by hypoxia, inflammation, mechanical stress, and nutrient limitation (61–63). Because ferroptosis relies heavily on cellular lipid, amino acid, and iron metabolic states, growing evidence indicates that effective prevention or intervention for ferroptosis in OA demands an in-depth understanding and modulation of these reprogrammed metabolic processes so as to curb OA progression at its source.

### 3.1 Lipid metabolic reprogramming

#### 3.1.1 PUFAs and lipid peroxidation

Lipid peroxidation is the central event in ferroptosis. When abundantly incorporated into membrane phospholipids, PUFAs greatly increase cellular susceptibility to ferroptosis due to their high autoxidation potential (18, 64). Under physiological conditions, cartilage maintains a balanced PUFA profile that supports membrane fluidity and lipid signaling. However, in OA, chronic inflammation and oxidative stress often dysregulate lipid metabolism, leading to increased synthesis and accumulation of n-6 PUFAs such as arachidonic acid and linoleic acid. These n-6 PUFAs are prone to peroxidation and have been implicated in promoting inflammation and cartilage degradation. In contrast, n-3 PUFAs like EPA and DHA exhibit anti-inflammatory and chondroprotective properties. The resulting imbalance elevates the content of oxidation-sensitive n-6 PUFA-containing phospholipids within cartilage membranes. Upon exposure to ROS or iron-catalyzed oxidative stress in OA joints, these phospholipids undergo lipid peroxidation, producing reactive aldehydes such as MDA and 4-HNE. These byproducts contribute to membrane disruption and may facilitate ferroptosis, depending on the local oxidative environment and PUFA composition (65, 66).

#### 3.1.2 Expression and regulation of key enzymes

Acyl-CoA synthetase long-chain family member 4 (ACSL4) and lysophosphatidylcholine acyltransferase 3 (LPCAT3) are key enzymes that modulate phospholipid synthesis and remodeling. The contribution of ACSL4 to ferroptosis hinges on its ability to attach CoA moieties to long-chain PUFAs, after which LPCAT3 or related enzymes esterify these long-chain PUFAs into lysophospholipids. Once PUFA-containing phospholipids become integrated into cell membranes, they are highly susceptible to peroxidation. If not eliminated by GPX4, the resultant accumulation of lipid peroxidation products drives ferroptosis. Both ACSL4 and LPCAT3 directly determine the PUFA content in cellular membranes and are thus essential for ferroptosis induction (67–69).

ACSL4 and LPCAT3 serve as central lipid metabolic regulators of ferroptosis, yet their functions appear to be disease and context dependent. In most ferroptosis-related conditions including hepatic steatosis, cardiomyopathy, spinal cord injury, and BPA-induced liver damage, both enzymes are simultaneously upregulated, promoting lipid peroxidation and enhancing ferroptotic signaling (70–73). However, in the context of OA, recent studies suggest a more nuanced regulatory landscape. ACSL4 is commonly upregulated in OA cartilage and synovial cells, expediting the activation and incorporation of PUFAs into membrane phospholipids and consequently heightening ferroptotic sensitivity (57, 74). In human OA cartilage, ACLT-induced models and IL-1β-stimulated chondrocytes, both ACSL4 and lipid peroxidation marker 4-HNE are elevated. Genetic knockdown or pharmacological inhibition of ACSL4 can partially ameliorate lipid peroxidation-induced chondrocyte damage and slow OA progression (75–77).

In contrast, the role of LPCAT3 in OA appears more variable. Recent bioinformatic analyses based on GEO datasets (GSE51588 and GSE55457) identified LPCAT3 as one of the ferroptosis-related differentially expressed genes significantly downregulated in OA tissues. Machine learning algorithms including SVM-RFE, LASSO, and Random Forest further confirmed LPCAT3 and PGD as potential diagnostic markers, and ROC curve analysis showed good discriminative performance. *In vitro* validation revealed that LPCAT3 expression was significantly reduced in IL-1β-stimulated chondrocyte models in a dose-dependent manner. CIBERSORT analysis linked LPCAT3 expression to distinct immune cell infiltration profiles, suggesting an immune-metabolic regulatory role (78).

Mechanistically, LPCAT3 is regulated not only at the transcriptional level but also through m6A methylation. In OA, downregulation of METTL3 decreases LPCAT3 mRNA enrichment and stability, contributing to its suppression. Functionally, LPCAT3 mitigates chondrocyte ferroptosis by preserving mitochondrial integrity, enhancing GSH levels, and upregulating GPX4. It also directly interacts with ACSL4 and negatively regulates its expression, thereby restraining lipid peroxidation. *In vivo*, knockdown of LPCAT3 aggravates OA progression and inflammation, while pharmacological inhibition of ACSL4 partially rescues these effects (76). Unlike in cancer where LPCAT3 cooperates with ACSL4 to promote ferroptosis, in OA it appears to play a protective role. Collectively, these findings support the notion that LPCAT3 serves not only as a core lipid remodeling enzyme in the ferroptosis cascade but also as a disease-specific modulator of immune-metabolic interactions in OA. Its downregulation contrasts with its ferroptosis-promoting role in other pathologies such as cancer, highlighting the context-dependent complexity of ferroptotic regulation. Nevertheless, current evidence is primarily derived from *in vitro* models and limited animal studies. Future investigations should incorporate high-resolution joint-tissue single-cell transcriptomics, long-term OA models, and clinically stratified cohorts to further elucidate the diagnostic utility and mechanistic function of LPCAT3 in OA pathogenesis.

#### 3.1.3 Phospholipid oxidation products and inflammatory signaling

Oxidized phospholipids generated through peroxidation, such as hydroxylated lipids and keto-lipids, not only compromise the integrity of the cell membrane directly but also increase membrane tension in tandem with enhanced lipid peroxidation. This heightened membrane tension activates Piezo1 and transient receptor potential (TRP) mechanosensitive ion channels, while simultaneously inactivating membrane Na+/K+-ATPases. The resultant ionic imbalance triggers changes in intracellular osmotic pressure, leading to cellular swelling—an important event in the execution of ferroptosis (79). When the concentration of the lipid peroxidation product 4-HNE is relatively low, it can augment IKB kinase (IKK) activity, promoting IKB phosphorylation. This results in the liberation of additional NF-KB, which upregulates proinflammatory genes and activates downstream NF-KB inflammatory pathways (80). Consequently, a “lipid-inflammation” vicious cycle arises in the OA joint microenvironment. Blocking or expediting the clearance of these lipid peroxidation products is therefore crucial for alleviating both OA-related inflammation and ferroptosis.

### 3.2 Amino acid metabolic reprogramming

#### 3.2.1 The central role of the glutamate-GSH pathway in ferroptosis

Ferroptosis is highly dependent on GPX4-mediated reduction of lipid peroxides, and reduced GSH serves as GPX4’s substrate. Glutamate, cysteine, and glycine are the three primary amino acids constituting GSH, with cysteine acquisition being the most pivotal (81). Under normal conditions, System Xc−, composed of the functional subunit SLC7A11 and the regulatory subunit SLC3A2, facilitates the exchange of extracellular cystine for intracellular glutamate, ensuring a sufficient supply of GSH precursors (82). When System Xc− function is impaired or SLC7A11 expression decreases, GSH synthesis becomes limited, and GPX4 activity can no longer be sustained, ultimately exacerbating ferroptosis (83). Hydrogen sulfide can protect cardiomyocytes by activating the SLC7A11/GSH/GPX4 axis and inhibiting ferroptosis (84). In OA chondrocytes, chronic exposure to mechanical overload and inflammatory stimuli such as interleukin-1β and tumor necrosis factor-α disrupts cellular redox homeostasis, rendering the glutamate–GSH antioxidant system susceptible to oxidative damage. In tumor cells, p53 has been well characterized as a promoter of ferroptosis through suppression of SLC7A11 expression and subsequent inhibition of cystine uptake. However, whether a similar p53–SLC7A11 regulatory mechanism contributes to ferroptosis susceptibility in OA chondrocytes remains unclear and requires further investigation (27, 85).

#### 3.2.2 The Met/cystine cycle and other amino acid pathways

Beyond the direct uptake of extracellular cystine, cells can also generate cysteine via the methionine (Met) cycle and transsulfuration pathway (86). These biochemical cycles in OA may be influenced by inflammation, oxidative stress, and fluctuations in enzyme activity. Additionally, amino acid pathways involving arginine, ornithine, and proline are tightly linked to extracellular matrix synthesis, nitric oxide (NO) signaling, and immune regulation, which may indirectly affect ferroptosis by modulating ROS production and amino acid availability (87–89).

#### 3.2.3 Amino acid derivatives and joint protection

Certain amino acid–derived small molecules, such as N-acetylcysteine (NAC) and taurine, have notable antioxidant and anti-inflammatory properties (90, 91). These molecules can replenish cellular reservoirs of reducing agents to some extent, thereby enhancing GPX4’s capacity to neutralize lipid peroxidation and exerting a potential protective effect on chondrocytes. In OA animal models, oral or local administration of NAC has been observed to mitigate cartilage damage and pain, closely tied to its role in sustaining GSH levels and inhibiting ferroptosis (92).

### 3.3 Iron metabolic reprogramming

#### 3.3.1 Alterations in proteins governing iron uptake, storage, and export

Ferroptosis is closely associated with dysregulated iron homeostasis, in which intracellular iron accumulation facilitates lipid peroxidation and cell death. In OA, iron overload has been shown to alter the expression of iron metabolism-related proteins. Specifically, chondrocytes exposed to ferric ammonium citrate (FAC) exhibit increased expression of FTH1 and reduced levels of transferrin receptor 1 (TfR1) and hepcidin, whereas ferroportin (FPN1) expression remains unchanged (38). Similar patterns of FTH1 upregulation and TfR1 downregulation have also been reported in primary murine chondrocytes under iron overload (93) and in cartilage tissue from OA models (94). Although systemic inflammation is known to modulate iron metabolism, such as interleukin-6 (IL-6) stimulating hepcidin synthesis and subsequently downregulating FPN1, this has been observed primarily in hepatic tissues (95). There is currently insufficient evidence to support whether this regulatory axis operates within cartilage. Moreover, no established strategies exist to locally modulate iron efflux through FPN1 or hepcidin in OA joints. Further investigation is warranted to determine whether targeting local iron export mechanisms could alleviate ferroptosis-related cartilage degeneration.

#### 3.3.2 Fenton reactions and the bidirectional effects of iron homeostasis imbalance

Free Fe²^+^ participates in the Fenton reaction, leading to the generation of hydroxyl radicals (·OH), which represents a crucial step in initiating the lipid peroxidation chain reaction (96). As local iron content in joints rises, cells become more prone to ferroptosis. Meanwhile, cells undergoing ferroptosis can release iron ions and oxidation byproducts, further disrupting iron homeostasis in neighboring cells, thereby exacerbating lesion spread.

#### 3.3.3 Local iron homeostasis and the joint microenvironment

Pathological changes such as increased vascular permeability or microbleeding in subchondral bone can also lead to local iron deposition. Proteomics data suggest that Ras homolog family member A (RhoA) may be associated with vascular permeability in subchondral bone and ferroptosis in OA. Analyses of clinical samples show that RhoA expression is markedly elevated in OA subchondral bone, aligning with the proteomics findings. Mechanistic studies indicate that RhoA exacerbates vascular permeability in OA by inhibiting endothelial cell adhesion proteins and actin filaments and by promoting endothelial ferroptosis (97). Single-cell data and clinical sample analyses further reveal that the iron content of synovial fluid and cartilage from OA patients is higher than that in healthy controls; patients with notably high iron levels sometimes report intensified pain and diminished joint function (98, 99). These observations underscore the importance of regulating local iron homeostasis to slow OA progression and reduce ferroptosis events.

### 3.4 Glucose and energy metabolic pathways

#### 3.4.1 Adaptive changes in glycolysis and oxidative phosphorylation in OA chondrocytes

Under normal conditions, chondrocytes primarily derive energy through anaerobic glycolysis. In OA, however, due to an inflammatory microenvironment and cellular stress, glycolytic flux often becomes further elevated to meet energy demands, while mitochondrial dysfunction may reduce oxidative phosphorylation efficiency (100). Although upregulated glycolysis can temporarily support cell survival, the resultant accumulation of lactate and H+ alters local pH, undermines cartilage matrix homeostasis, and influences both amino acid and lipid metabolic pathways (101).

#### 3.4.2 Effects of glucose metabolic reprogramming on lipid and amino acid metabolism

Reprogrammed glucose metabolism primarily impacts two areas. First, its interplay with lipid metabolism: surplus pyruvate generated through glycolysis is converted to acetyl-CoA, which can enter lipid biosynthetic pathways and promote the synthesis of fatty acids, particularly PUFAs. Hence, an active glucose metabolism partly amplifies the substrate pool for lipid peroxidation (102). Second, its interaction with amino acid metabolism: intermediates of glucose metabolism and the tricarboxylic acid (TCA) cycle provide carbon skeletons for amino acid synthesis and transformation, while also modulating NADH levels, thereby influencing GSH production (103). Abnormalities in glucose metabolism compromise GPX4’s antioxidant capacity and disrupt cysteine cycling, indirectly driving ferroptosis.

#### 3.4.3 Energy status and oxidative stress balance

In OA lesions, synovial inflammation and cartilage degeneration reduce local nutrient availability and oxygen levels, compelling cells to adapt their energy-generating modes (104). Energy molecules such as ATP and NADPH not only govern cellular proliferation and protein synthesis but also play integral roles in oxidative damage repair and maintenance of the antioxidant system. Consequently, dysregulated glucose metabolism frequently correlates positively with uncontrolled ROS production and heightened ferroptosis risk.

In OA, cells and tissues undergo extensive metabolic reprogramming in response to chronic inflammation and mechanical stress. Excessive lipid reprogramming increases the proportion of PUFAs within phospholipids, providing the “fuel” for ferroptosis; amino acid imbalances undermine the GPX4 antioxidant “defense”; disrupted iron metabolism results in local iron overload and augmented Fenton reactions; and aberrant glucose and energy metabolism furnishes the substrates and energy that drive all these processes forward. A deeper understanding of these interconnected metabolic networks clarifies the critical role of ferroptosis in OA progression and lays a robust foundation for integrated therapeutic strategies targeting both metabolic reprogramming and ferroptosis.

## 4 Immune and metabolic crosstalk in the regulation of ferroptosis

Beyond metabolic changes, immune components significantly influence ferroptotic events in OA. In this section, we detail how immunoinflammatory processes intersect with ferroptosis pathways.

OA is a degenerative disorder affecting the entire joint structure. Traditionally, it has been attributed to mechanical-stress-related cartilage degeneration. However, a growing body of evidence reveals a pronounced immunoinflammatory phenotype in OA, wherein macrophages, T/B lymphocytes, and inflammatory factors such as IL-1β, TNF-α, and IL-6 play a critical role in the local microenvironment (105). Within the metabolic reprogramming and ferroptosis mechanisms discussed earlier, these immune cells and inflammatory mediators modulate iron homeostasis and lipid peroxidation, among other metabolic pathways. Conversely, the release of cellular components or inflammatory signals following ferroptotic cell death can amplify immune responses, establishing a self-perpetuating “immunometabolic crosstalk” that may accelerate lesion progression and exacerbate tissue damage in OA.

### 4.1 The role of immune cells in the local microenvironment of OA

Macrophages are among the most prominent immune cells within the OA synovium. They can differentiate into a proinflammatory (M1) or anti-inflammatory (M2) phenotype depending on local cues. M1 macrophages, typically activated by IFN-γ, are characterized by the production of IL-1β, TNF-α, IL-6, and iNOS, and are closely associated with synovial inflammation and tissue destruction (106). Inflammatory M1 macrophages also exhibit distinct iron-handling features, including increased expression of ferritin (FtH, FtL) and hepcidin, coupled with reduced FPN1, promoting intracellular iron retention (95, 107, 108). This iron-rich profile may exacerbate oxidative stress and sensitize tissues to ferroptosis. Interestingly, external iron supplementation has been shown to inhibit M1 polarization by downregulating proinflammatory mediators and iNOS expression through STAT1 inhibition, without impairing phagocytic capacityM1 (108). These findings suggest a dual role for iron in M1 macrophages: while endogenous iron sequestration may facilitate ferroptosis, excess extracellular iron may conversely suppress inflammatory activation.

In contrast, M2 macrophages generally favor iron export and exhibit anti-inflammatory functions. However, in OA, M2 numbers are limited and may shift toward an M1-like phenotype under sustained inflammatory stimuli, reducing their capacity to maintain iron homeostasis (109, 110).Together, these insights support the hypothesis that iron metabolism in M1 macrophages not only contributes to ferroptosis susceptibility in OA, but also represents a potential immunometabolic checkpoint for therapeutic intervention.

T lymphocytes in OA frequently assume an activated state. Helper or cytotoxic T cells secrete factors such as interferon-γ (IFN-γ) and IL-17 that compromise cartilage integrity (111, 112). T-cell surface molecules or secreted factors may also regulate the expression of lipid-metabolic enzymes such as ACSL4, thereby indirectly fostering ferroptosis (113).

Although dendritic cells (DCs) and natural killer (NK) cells are less abundant in OA synovium compared with macrophages and T cells, they can still influence synovitis severity and iron homeostasis by secreting inflammatory mediators or cooperating with T cells. NK cells infiltrate the synovium and joint fluid of OA patients, with both their number and function fluctuating as the disease progresses. NK cells can eliminate abnormal joint cells or pathogens via perforin and granzymes, and they secrete cytokines such as IFN-γ and TNF-β to regulate immunity and inflammation (1, 114, 115). Some studies have noted that although NK cells can clear certain damaged chondrocytes or pathogens in early OA, they also release ROS under highly inflammatory conditions, aggravating oxidative stress and indirectly activating ferroptosis pathways (116).

### 4.2 Regulation of ferroptosis by inflammatory cytokines and immune signaling

#### 4.2.1 Classic proinflammatory cytokines

IL-1β can induce the synthesis of matrix-degrading enzymes MMP-13 and ADAMTS-5 by chondrocytes and synovial cells (117). In gastric cancer, however, IL-1β has been shown to induce the mitochondrial translocation of the acetyltransferase PCAF, which acetylates the mitochondrial inner membrane protein NNT at K1042. This modification enhances NNT’s affinity for its substrate NADP+, promoting NADPH synthesis, maintaining iron–sulfur cluster homeostasis, lowering intracellular ferrous iron levels and lipid peroxidation, and thereby protecting tumor cells from ferroptosis (118).

Apart from exacerbating local inflammation, TNF-α can activate the NF-KB and MAPK signaling pathways, increasing cellular susceptibility to oxidative stress and weakening GPX4 and other antioxidant enzymes, thereby promoting further accumulation of lipid peroxidation byproducts and markedly elevating ferroptosis risk (119). Studies have also indicated that elevated TNF-α and dysregulated iron homeostasis often reinforce each other in tumor or inflammation models, collectively driving cell damage and death (120).

As noted earlier, IL-6 is often significantly upregulated in the synovial fluid and serum of OA patients. By increasing the expression of hepcidin or suppressing FPN, IL-6 raises intracellular free iron levels, amplifying lipid peroxidation under iron-rich conditions and heightening ferroptosis (40). Furthermore, IL-6 may regulate metabolic reprogramming in chondrocytes, particularly affecting lipid and amino acid metabolism, through activation of the JAK STAT pathway, thereby influencing the synthesis and balance of GSH and NADPH. These mechanisms highlight IL-6’s dual role in immunity and ferroptosis: on one hand, it can promote iron overload; on the other, it can intensify oxidative stress by altering metabolic pathways (121).

Although IFN-γ is not one of the three most frequently implicated proinflammatory cytokines in OA, its role in ferroptosis regulation warrants attention. Evidence suggests that IFN-γ suppresses System Xc− transcription by activating the JAK-STAT-IRF1 pathway, which depletes intracellular GSH stores, intensifies lipid peroxidation, and induces ferroptosis in various tumor and immune cells. In erastin- or RSL3-induced ferroptosis in hepatocellular carcinoma cells, IFN-γ triggers the JAK-STAT-IRF1 axis and inhibits transcription of the System Xc− subunits SLC3A2 and SLC7A11 (122, 123). In conjunction with other cytokines such as TNF-α and IL-6, this forms a critical axis impacting ferroptosis in inflammatory and tumor immune environments.

#### 4.2.2 Other key signaling pathways

NF-KB signaling lies at the nexus of many inflammatory cytokines and immune receptors, thus exerting bidirectional effects on ferroptosis. In OA and other inflammation-related diseases, NF-KB activation commonly leads to decreased transcription of antioxidant factors such as GPX4, NQO1, and HMOX1, thereby promoting ferroptosis (124, 125). In RSL3-induced glioblastoma cell models, excessive activation of NF-KB represses ATF4 and SLC7A11, causing overproduction of lipid peroxidation byproducts and exacerbating ferroptosis (126). However, loss of leukemia inhibitory factor receptor (LIFR) positively regulates NF-KB activation and enhances the secretion of lipocalin-2 (LCN2) to sequester extracellular iron. Phosphorylated p65 promotes the transcription of LCN2, impeding iron influx and decreasing cellular susceptibility to ferroptosis inducers (120).

The Nrf2/HO-1 pathway plays a vital role in antioxidative and iron-homeostatic responses, exhibiting a well-documented “double-edged sword” effect in ferroptosis regulation. On one hand, moderate HO-1 expression can degrade heme to reduce free iron, inhibit NF-KB activation, and alleviate inflammation and oxidative stress (127). On the other hand, overexpression of HO-1 can lead to excessive free iron release, promoting Fenton reactions and potentially exacerbating ferroptosis under specific conditions (128). Furthermore, Nrf2/HO-1 interacts with the NF-KB and JAK-STAT pathways in various contexts: Nrf2/HO-1 can reduce NF-KB phosphorylation and thereby dampen inflammation, whereas proinflammatory cytokines such as TNF-α and IL-6, released following NF-KB activation, upregulate HO-1 in a feedback loop (125, 129). Meanwhile, the JAK-STAT-driven inflammatory mediators IL-6 and IFN-γ upregulate hepcidin, suppressing iron export and causing intracellular iron accumulation. Nrf2/HO-1, by enhancing iron storage and neutralizing iron toxicity, restricts lipid peroxidation (130). In this context, inflammatory cytokines and immune cell secretions function as a “regulatory valve.”

In addition to NF-KB and Nrf2/HO-1, the NLRP3 inflammasome and cGAS-STING pathways also play distinctive roles in ferroptosis–inflammation crosstalk. The NLRP3 inflammasome is activated by lipid peroxidation byproducts such as 4-HNE and MDA produced during ferroptosis, triggering caspase-1-dependent maturation of IL-1β/IL-18, which amplifies inflammation and establishes a vicious cycle (127, 131). Meanwhile, cGAS-STING senses mitochondrial DNA (mtDNA) released from damaged mitochondria during ferroptosis and drives the production of type I interferons (IFN-I), thereby regulating expression of TfR1 and recruiting immune cells to intensify tissue injury (132, 133). These two pathways cooperate in several ways: IFN-I generated by cGAS-STING heightens NLRP3 sensitivity, and NLRP3-dependent IL-1β upregulates cGAS via NF-KB, creating a positive-feedback loop. STING agonists and ferroptosis inducers jointly activate NLRP3 (134, 135). Hence, targeting NLRP3 or STING could potentially interrupt the reciprocal cycle between inflammation and ferroptosis.

### 4.3 Mutual promotion or inhibition within the immunometabolic network and ferroptosis

#### 4.3.1 Impact of immunocyte metabolic reprogramming on ferroptosis susceptibility

**Figure 2:**
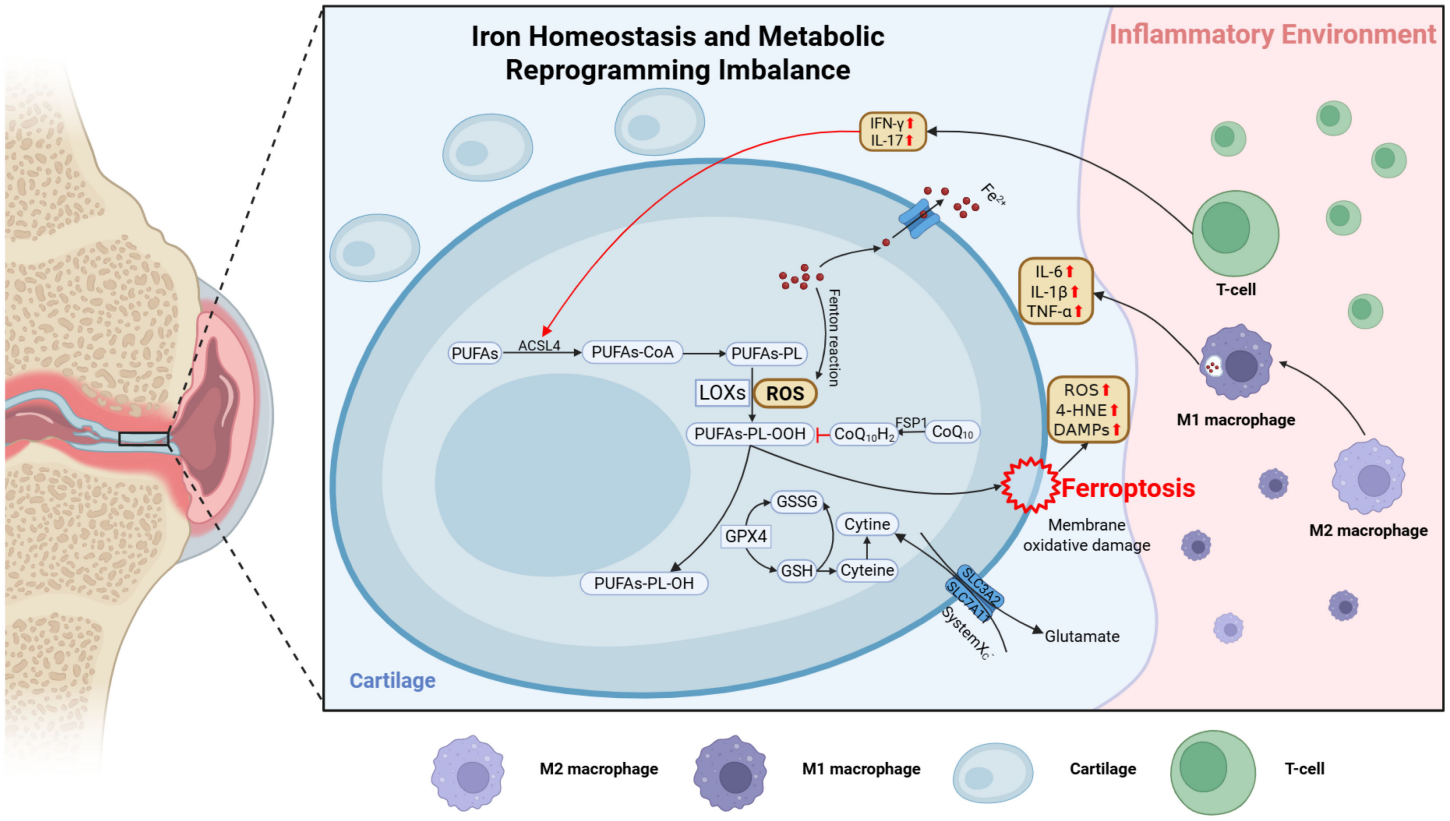
Schematic illustration of ferroptosis regulation in the OA joint microenvironment. Mechanical stress and inflammatory cytokines (e.g., IL-1β, TNF-α, IL-6) disrupt lipid, amino acid, and iron metabolism in chondrocytes, promoting ferroptosis. In lipid metabolism, ACSL4 is upregulated and promotes PUFA incorporation into membrane phospholipids, driving lipid peroxidation and ferroptosis. Inhibition of System Xc− (SLC7A11) reduces GSH synthesis and impairs GPX4 function. Ferroptotic cells release 4-HNE and DAMPs, which activate M1 macrophages and T cells to secrete IL-1β, IFN-γ, IL-17, and hepcidin, amplifying oxidative damage. This immunometabolic feedback loop accelerates cartilage degradation, synovitis, and subchondral bone remodeling. The figure was created with BioRender (https://biorender.com/).

Under chronic inflammatory conditions, immune cells themselves undergo metabolic reprogramming. For instance, activated macrophages and T cells often exhibit heightened glycolysis and altered mitochondrial function, affecting the synthesis of NADPH and GSH (136, 137). When immune cells adopt a proinflammatory metabolic phenotype, for example M1-dominant macrophages, elevated ROS production and disrupted iron homeostasis often predispose both these cells and surrounding tissues to ferroptosis. Conversely, guiding immune cells toward an anti-inflammatory phenotype, such as M2-skewed macrophages, can enhance lipid clearance and reduce iron overload in OA joints, potentially lowering ferroptosis incidence (109) (**Figure 2**).

#### 4.3.2 Amplification of immune responses by released intracellular components following ferroptosis

When ferroptosis leads to the rupture of chondrocytes or synovial cells, the oxidized lipid byproducts, iron ions, and damage-associated molecular patterns (DAMPs) that leak from these cells are detected by nearby immune cells. This recognition step triggers further cytokine release and inflammation, thereby perpetuating an “inflammation–ferroptosis–inflammation” positive feedback loop (138). Such feedback not only accelerates cartilage degeneration but also complicates potential therapeutic interventions.

### 4.4 Interactions between cartilage and synovium

#### 4.4.1 Interplay between cytokines released by chondrocytes and synovial cells

When cartilage is damaged, chondrocytes release proteoglycan fragments, type II collagen fragments, and diverse injury-associated factors that synovial cell surface pattern recognition receptors (PRRs) can detect, prompting synovial hyperplasia and proinflammatory cytokine secretion (1, 139). Conversely, synovial-derived cytokines such as IL-1β and TNF-α further stimulate chondrocytes, aggravating lipid peroxidation and ferroptosis (140, 141).

#### 4.4.2 Synovitis, ferroptosis, and iron homeostasis imbalance

Although synovitis is not the primary lesion in OA, its role as a possible “inflammatory amplifier” cannot be overlooked (142). Persistent synovitis can induce VEGF production, promoting neovascularization and increased vascular permeability (143), which may facilitate the influx of iron into the joint cavity and subchondral bone and recruit additional immune cells, thereby intensifying local oxidative stress and inflammatory cascades (144).

During OA, synovial tissue exhibits an inflammatory response, releasing IL-1, IL-6, TNF-α, and other cytokines and chemokines that attract peripheral immune cells such as macrophages, neutrophils, and T cells to the synovial membrane (145, 146). TNF-α can induce synovial cells to express intercellular adhesion molecule-1 (ICAM-1), making it easier for immune cells to adhere and transmigrate across endothelial cells into the synovium (147).

As immune cells gather and become activated in the inflamed synovium during OA, ROS production significantly increases. When macrophages and other immune cells engulf pathogens or damaged tissue debris, they generate large amounts of ROS, such as superoxide anions, hydrogen peroxide, via respiratory burst (148). An overabundance of ROS disrupts intracellular redox balance, triggering oxidative stress and elevating susceptibility to ferroptosis. Elevated ROS levels in OA synovium have been confirmed experimentally, where mean fluorescence intensity of ROS is markedly higher than in healthy controls, indicative of pronounced oxidative stress in OA joints (149).

The cytokines and oxidative stress byproducts arising from synovitis further activate synovial cells and chondrocytes to release additional inflammatory mediators and catabolic enzymes. These mediators, in turn, recruit more immune cells, establishing a vicious cycle that continually amplifies inflammatory cascades. IL-1β and TNF-α stimulate chondrocytes and synovial cells to secrete MMP-1, MMP-3, and MMP-13, which degrade collagen and proteoglycans in the cartilage matrix, worsening cartilage damage (150, 151). Concurrently, oxidative stress can trigger NF-KB signaling, upregulating inflammation-related genes and exacerbating ferroptosis (125).

Pathologically, cartilage and synovium are mutually dependent and mutually reinforcing in the context of ferroptosis in OA. Therefore, therapies targeting only cartilage or synovium may be insufficient; a multilayered approach that concurrently inhibits synovial inflammation, modulates immunometabolic processes, and safeguards chondrocytes from ferroptosis-induced injury may yield more robust clinical benefits.

In summary, immunometabolic crosstalk is a pivotal feature of OA progression and a critical component of ferroptosis regulation. Activated immune cells, particularly macrophages and T/B lymphocytes, profoundly influence iron homeostasis and lipid peroxidation through NF-KB, Nrf2/HO-1, and other signaling pathways. Conversely, ferroptosis-related release of oxidized byproducts and iron ions feeds back to escalate inflammation and further damage cartilage and synovium, establishing a vicious cycle. To effectively target ferroptosis in OA, both the induction of ferroptosis by immune mediators and the adaptive changes of metabolic reprogramming must be considered. A multifaceted intervention at the “immunometabolic” intersection of synovium and cartilage may maximize structural and functional improvements for OA-affected joints.

## 5 Intervention strategies targeting ferroptosis in OA and future perspectives

Having delineated the pathophysiological links between OA and ferroptosis, we now examine current and emerging therapeutic approaches that aim to block or mitigate ferroptosis and potentially preserve joint integrity.

Drawing upon prior discussions on the relationship between OA and ferroptosis, blocking or slowing ferroptosis holds promise for protecting cartilage, reducing synovitis, and improving joint function. Given the highly complex mechanisms of ferroptosis and the fact that OA pathology often involves multiple dysregulated pathways, an integrated, multifaceted treatment approach is required. In this section, we explore potential therapeutic strategies reported to date, discussing their practical applications and limitations in OA, and then offer prospects for future investigations.

### 5.1 Intervention strategies

#### 5.1.1 Targeting iron homeostasis

Iron chelators such as deferoxamine, deferiprone, and deferasirox bind free ferrous iron (Fe2+), reducing substrate availability for the Fenton reaction and thereby curtailing the lipid peroxidation cascade (152). Animal studies suggest that local or systemic use of deferoxamine may significantly alleviate OA cartilage degradation and inhibit ferroptosis markers in chondrocytes by activating the Nrf2 pathway (54). However, prolonged use of iron chelators might disrupt normal hematopoiesis and iron utilization, necessitating further safety and efficacy assessments in clinical settings (**Table 1**) (168, 169).

**Table 1:** Potential ferroptosis-targeted therapeutic strategies in OA and key research findings.

Strategy/Drug	Mechanism	Models	Molecular Targets	Key Effects	References
**1. Iron Chelators**(DFO, DFP, DFX)	Bind free iron; reduce Fenton reactions	IL-1β-treated chondrocytes; DMM/ACLT	Iron homeostasis; GPX4	Lower ROS/MDA; alleviate cartilage damage	(41, 138–140)
**2. TfR1 Inhibition or FPN Upregulation** (Ferstatin II, SM04690@ApoF, BCA)	Decrease iron uptake; enhance iron export	Oxidative/inflammatory chondrocytes; surgical OA models	TfR1; FPN	Lessen iron overload; attenuate lipid peroxidation	(141–143)
**3.Antioxidant and GPX4 Enhancement**(Icariin, Kukoamine A, BoNT/A, NAC, Vitamin K2, CoQ10)	Boost GPX4; supply antioxidants	IL-1β, erastin, RSL3 models; DMM/ACLT	SLC7A11/GPX4; SIRT1/GPX4	Reduce ROS; protect cartilage	(144–151)
**4. Lipid Peroxidation Inhibitors** (Ferrostatin-1, Liproxstatin-1)	Capture lipid radicals; halt PUFA chain	IL-1β or TBHP-stimulated cells; DMM/ACLT	Lipid peroxidation intermediates	Decrease chondrocyte death; improve OARSI scores	(40, 57, 152)
**5. Nrf2/HO-1 Regulation** (Paeonol, Protopine, Sappanone A, Genipinic Acid)	Activate Nrf2; caution excessive HO-1	IL-1β/TBHP chondrocytes; DMM/ACLT	Nrf2/HO-1; GPX4	Inhibit ferroptosis/inflammation; moderate iron	(7, 113, 114, 153–155)
**6. Immune Pathway Blockade** (Anti–IL-1β, Anti–TNF-α, IL-6 Inhibitors)	Suppress proinflammatory cytokines	Mostly RA trials; early OA studies	NF-KB; p53	Reduce local inflammation; lower iron imbalance	(13, 106, 156–158)
**7. Gene-Based Therapies** (siRNA, Genome Editing, m^6^A Modulation)	Silence ACSL4/p53; stabilize GPX4 mRNA	siRNA chondrocytes; gene-modified mice	ACSL4; p53; METTL14	Curtail PUFA–ROS; protect ECM	(159–163)
**8. MSC Transplantation/Exosomes**(BMSC, SMSC)	Paracrine factors/miRNAs modulate ferroptosis	OA animal models (intra-articular); co-culture	Iron homeostasis; immune regulation	Enhance cartilage repair; lower ROS/inflammation	(164–167)
**9. Natural Compounds & Combination Therapy**(Metformin + ACSL4 Inhibition, Flavonoids, Extracts)	Adjust glycolipid metabolism; limit iron	Cell and animal studies (AMPK activation, flavonoids)	ACSL4; p53; GPX4	Decrease PUFA synthesis; anti-oxidation synergy	(44, 57–59)
**10. Nanocarriers/Hydrogels**(Se/Ga Nanoparticles, Micelle–Hydrogel)	Targeted drug delivery; prolong retention	OA models (intra-articular); drug-loaded materials	TfR1; Nrf2/GPX4	Dampen inflammation; safeguard cartilage	(141, 142, 155)

*DMM, Destabilization of the Medial Meniscus; ACLT, Anterior Cruciate Ligament Transection; TBHP, tert-butyl hydroperoxide (an oxidative stress inducer).

Bold values indicate overarching categories or representative strategies, under which specific drugs or interventions are listed.

Modulating TfR1 or enhancing FPN function can also curb ferroptosis by adjusting iron homeostasis. Inhibiting TfR1 reduces cellular iron uptake from the outset, lowering ferroptosis risk. Related inhibitors, explored extensively in oncology, remain in early-stage OA studies. For example, the TfR1 inhibitor Ferstatin II significantly suppresses TfR1 activity *in vitro* and *in vivo*, improving cartilage degradation. In post-DMM mouse models of traumatic OA, TfR1 expression is elevated in cartilage and fosters inflammation. Silencing TfR1 not only reduces chondrocytic iron content and oxidative stress but also promotes mitophagy and inhibits the mtDNA/cGAS/STING inflammatory axis, thereby breaking the cycle between ferroptosis and immune activation. A hydrogel microsphere with micelle microfluidic properties contains a ketone enol thiol-bridged nanoscale secondary structure, which downregulates TfR1 expression through metal chelation and increases the expression of CDGSH iron sulfur domain-containing protein 1 (CISD1), thereby restoring mitochondrial iron homeostasis and promoting cartilage repair (170).

In the realm of natural compounds, biochanin A (BCA), extracted from Astragalus, protects against bone loss by inhibiting TfR1 and enhancing FPN, thereby reducing intracellular iron levels. It also targets the Nrf2/System Xc−/GPX4 pathway to remove free radicals and prevent lipid peroxidation. Micro-CT and histological staining of knee joints show that BCA prevents cartilage iron deposition and reduces KOA severity in iron-overloaded mice, suggesting new therapeutic avenues for KOA (171).

#### 5.1.2 Antioxidants and inhibitors of lipid peroxidation

GPX4 activators and ROS scavengers effectively suppress ferroptosis. Since GPX4 is critical for neutralizing lipid peroxides, enhancing GPX4 function or ensuring an ample supply of its substrate GSH can significantly lower ferroptosis incidence. Several small molecules and protein-based compounds are under development to modulate GPX4 enzyme activity. Icariin (ICA) can mitigate ferroptosis in OA rat models both *in vivo* and *in vitro*. *In vivo*, ICA treatment partially restored type II collagen and GPX4 expression, reduced ECM degradation in chondrocytes, and restrained chondrocyte ferroptosis, alleviating OA joint damage. *In vitro*, ICA effectively attenuated the IL-1β induced suppression of SLC7A11 and GPX4 expression in SW1353 cells, restored intracellular levels of ROS, lipid peroxides, and MDA, and significantly reduced erastin induced ferroptosis. By contrast, ICA exerted no protective effect in GPX4-silenced SW1353 cells, indicating that the drug ameliorates ferroptosis and cartilage injury via the SLC7A11/GPX4 pathway (153). Kukoamine A, a bioactive compound isolated from Lycium chinense, inhibits chondrocyte inflammation and ferroptosis via the SIRT1/GPX4 pathway, thus slowing OA progression (154). Botulinum neurotoxin type A (BoNT/A), an exotoxin from Clostridium botulinum, reversed the downregulation of type II collagen and the upregulation of MMP-13 induced by LPS in chondrocytes, alleviating LPS-induced chondrocyte toxicity, activating the SLC7A11/GPX4 antiferroptotic system, and enhancing mitochondrial function. *In vivo*, intra-articular BoNT/A administration reduced cartilage degradation in OA rats (172). Moreover, various ROS scavengers, including N-acetylcysteine (155), vitamin K2 (156), and coenzyme Q10 (157–159), have demonstrated joint-protective effects in animal models and *in vitro* experiments.

Inhibiting lipid peroxidation can suppress ferroptosis through two primary mechanisms. First, compounds such as Lip-1 and Fer-1 directly interrupt the lipid radical chain reaction, markedly attenuating ferroptosis (53). Animal research indicates that these agents can lessen articular cartilage injury and inflammation (160). Second, modulating key lipid-metabolizing enzymes, especially ACSL4, reduces PUFA incorporation into membrane phospholipids, lowering the substrate pool for lipid peroxidation and decreasing ferroptosis susceptibility. In OA models, ACSL4 inhibition dramatically decreases ferroptosis-associated lipid peroxidation (57, 74, 77). Metformin, via the AMPK/ACC pathway, reshapes lipid utilization and reduces ACSL4 and 4-HNE positivity, thereby improving chondrocyte resistance to ferroptosis (74). In a rat OA model, pioglitazone-induced activation of PPARγ not only alleviated OA symptoms but also curbed expression of the ferroptosis marker ACSL4. Although LPCAT3 is also involved in ferroptosis by mediating phospholipid re-esterification, studies reveal an interaction between LPCAT3 and ACSL4 proteins; upregulated LPCAT3 suppresses ACSL4 in *in vitro* models, inhibiting cartilage chondroptosis and improving OA (76). Elevating LPCAT3 mRNA levels can mitigate chondrocyte ferroptosis in OA, and its enzymatic activity correlates with local inflammatory levels. Methylation-mediated suppression of LPCAT3 mRNA expression aggravates OA in mouse models (76).

Excessive lipid metabolic reprogramming in OA significantly raises ferroptosis risk, so targeting lipid synthesis, transport, and degradation represents a promising upstream approach to reducing ferroptotic damage. Rational modulation of PUFA biosynthesis, including arachidonic and linoleic acids, may quell excessive inflammatory responses and concurrently optimize the metabolic environment for antioxidant defense (161). PLA2G6 participates in phospholipid remodeling (the Lands cycle) by synthesizing new phospholipids, altering membrane phospholipid composition and thus membrane fluidity and stability, which in turn affects ferroptosis sensitivity (162). Phospholipase PLA2G4 can limit lipid peroxidation and suppress ferroptosis in KRAS-mutant lung cancer (163). High activity of MBOAT1 and MBOAT2 uses monounsaturated fatty acids (MUFAs) rather than PUFAs for acylation of lysophospholipids, thus lowering the PUFA content in phospholipids and ferroptosis susceptibility (173).

#### 5.1.3 Modulating immune and inflammatory pathways

Targeting key regulators such as NF-KB and Nrf2 can not only reduce proinflammatory cytokine production but also indirectly rebalance iron homeostasis. For instance, vitamin K2 (VK2) mitigates cartilage injury in an *in vivo* rat OA model induced by ACLT and in an *in vitro* model of oxidative damage in chondrocytes caused by tert-butyl hydroperoxide (TBHP). It suppresses ECM degradation, counteracts RSL3 mediated inhibition of GPX4, increases intracellular GSH levels and the GSH/GSSG ratio, reduces MDA content, and inhibits activation of the MAPK/NF-KB pathways, thereby protecting chondrocytes from ferroptosis (156).

Similarly, activating the Nrf2/HO-1 axis to enhance cellular antioxidant gene expression can hamper ferroptosis, although the potentially excessive iron release from high HO-1 expression must be considered (128). Further research is needed to validate Nrf2 activation in OA. Protopine (PTP) significantly reduces inflammation and cytolysis in primary murine chondrocytes treated with TBHP by activating the Nrf2 pathway and inhibiting ferroptosis; intra-articular PTP administration upregulates GPX4 and confers antiferroptotic and anti-inflammatory effects on chondrocytes, thereby protecting joint cartilage (174). Many natural products likewise show promise for OA treatment, especially regarding ferroptosis in chondrocytes. Paeonol, the major bioactive compound in peony bark, is a natural free radical scavenger with antioxidant, anti-inflammatory, and cartilage-protective properties. Experiments indicate that paeonol markedly impedes IL-1β-induced ferroptosis and inflammation in chondrocytes, partly through the AMPK/Nrf2/GPX4 axis (8). Genipinic acid (GPA), extracted from Eucommia ulmoides, has also been verified *in vitro* and *in vivo* to alleviate DMM-induced OA. GPA attenuates IL-1β induced ferroptosis by reducing the levels of MDA, intracellular iron, and ROS, while simultaneously upregulating the expression of GSH, GPX4, and ferritin. GPA increases Nrf2 and HO-1 levels; conversely, an Nrf2 inhibitor abolishes its anti-inflammatory and antiferroptotic effects (164). Sappanone A similarly combats inflammation and ECM degradation in OA by attenuating chondrocyte ferroptosis through the SIRT1/Nrf2 pathway (165).

Overall, Nrf2-targeted therapies remain at an experimental stage, necessitating further drug development for clinical use.

Antagonists or neutralizing antibodies against IL-1β, TNF-α, and IL-6, such as IL-1Ra and anti-TNF-α, have been extensively studied in OA. These agents, by curbing the inflammatory cascade, may also help restore iron homeostasis and oxidative balance, thereby lessening ferroptosis. Although such strategies have exhibited favorable outcomes in rheumatoid arthritis, their efficacy in OA remains debated, necessitating additional clinical trials (166, 167, 175).

#### 5.1.4 Gene therapy and genome editing

Recent advances in gene therapy and genome editing offer promising approaches to modulate ferroptosis in OA by targeting critical regulators such as GPX4, SLC7A11, and epigenetic modifiers. For instance, METTL14-mediated m^6A methylation accelerates GPX4 mRNA degradation, thereby enhancing ferroptosis susceptibility in IL-1β-stimulated chondrocytes and OA cartilage. Knockdown of METTL14 by siRNA significantly reduces GPX4 mRNA m^6A modification, restores GPX4 expression, alleviates ECM degradation, and ultimately inhibits chondrocyte ferroptosis *in vitro* (176). Similarly, lncRNA MEG3 mitigates chondrocyte ferroptosis through a competing endogenous RNA (ceRNA) mechanism, sponging miR-885-5p to enhance the expression of SLC7A11, thereby reducing lipid peroxidation and iron-dependent cell death induced by erastin (177). These studies underscore the therapeutic potential of both protein-coding and noncoding RNAs as targets in OA ferroptosis intervention.

However, efficient and targeted intra-articular gene delivery remains a considerable technical challenge, primarily due to the dense extracellular matrix (ECM) of cartilage, rapid clearance of joint fluid, and limited cellular uptake. Lentiviral vectors (LV), adeno-associated virus (AAV), and lipid nanoparticles (LNP) are among the most studied delivery systems for intra-articular applications. LV vectors exhibit robust transduction efficiency in cultured chondrocytes but face safety concerns regarding potential insertional mutagenesis and immunogenicity. In contrast, AAV vectors, especially self-complementary AAV2 (scAAV2), have demonstrated superior penetration and stable gene expression in intact joint tissues. In rabbit models, intra-articular injection of scAAV2 encoding interleukin-1 receptor antagonist (IL-1Ra) yielded sustained therapeutic protein expression in both inflamed and normal joints for at least two weeks, with minimal immune activation or joint pathology (178). Nevertheless, achieving therapeutic efficacy with AAV often requires high viral titers, raising potential issues of dose-dependent cytotoxicity, immune responses, and eventual transgene silencing (179).

In order to improve the effect of gene therapy in regulating ferroptosis in OA, many studies in recent years have been devoted to optimizing the delivery system to overcome the complexity and barriers of the intra-articular environment (180). First, the engineering of vector capsids and the selection of serotypes are widely used to enhance the transduction efficiency of cartilage tissue. Kyöstio-Moore et al. systematically compared the transduction distribution of AAV1, 2, 5 and 8 serotypes in the knee joints of mice and found that different AAV serotypes showed different tissue tropisms in tissues such as synovium, cartilage and subsynovial bone (181). Similarly, Watson et al. reported that AAV can effectively transduce chondrocytes in the horse OA model. Although the transduction rate of synovial cells is slightly higher, the expression of cartilage tissue still has therapeutic potential (182).

Preconditioning tissues with mild inflammation or matrix disruption has also been shown to enhance viral uptake. IL-1β, for example, increases AAV transduction efficiency in human synovial cells, suggesting that the OA microenvironment may be leveraged to improve delivery (183). In addition to physical enhancement, transcriptional control through inflammation-responsive promoter systems such as those regulated by NF-KB can ensure greater transgene specificity. Pan et al. reported elevated AAV vector expression during inflammatory episodes, while synthetic promoters responsive to proinflammatory signals provide a promising platform for integrating CRISPR-Cas9 and other editing tools (184, 185).

Finally, combining vectors with biocompatible scaffolds or hydrogels may extend intra-articular retention and promote localized expression. Evans et al. highlighted that the rapid turnover of synovial fluid poses a challenge to sustained expression, underscoring the importance of incorporating physical carriers to protect and localize gene payloads (180).

In summary, gene therapy directed at ferroptosis regulators presents a promising strategy for OA intervention. Nonetheless, achieving effective and durable outcomes will require coordinated advances in vector design, targeting specificity, and intra-articular delivery technologies.

#### 5.1.5 Stem cell and exosome-based therapies

Mesenchymal stem cells (MSCs) have emerged as promising agents in OA therapy due to their immunomodulatory, antioxidative, and chondrogenic capacities. Beyond direct transplantation, increasing attention has shifted toward MSC-derived exosomes (MSC-Exos) as acellular, nanoscale delivery vehicles capable of regulating ferroptosis and joint degeneration through paracrine mechanisms (186).

Multiple studies have demonstrated that MSC-Exos attenuate ferroptosis in chondrocytes by targeting key molecular axes. For instance, bone marrow MSC-derived exosomes (BMSC-Exos) downregulate METTL3 expression, disrupt the m6A-mediated stabilization of ACSL4 mRNA, and ultimately reduce intracellular Fe²^+^, ROS, and lipid peroxidation. These changes correlate with increased GSH levels and decreased ACSL4 expression, thus mitigating ferroptotic cell death in IL-1β–stimulated chondrocytes and preserving cartilage integrity (187).

Noncoding RNAs also play a pivotal role. Exosomal lncRNA SNHG7 inhibits IL-1β-induced ferroptosis by sponging miR-485-5p and upregulating FSP1, a known ferroptosis suppressor, thereby restoring GPX4 levels and suppressing lipid peroxidation (188). Overexpression of miR-485-5p reverses this effect, confirming the functional specificity of this regulatory axis.

Similarly, MSC-Exos regulate the GOT1/CCR2/Nrf2/HO-1 axis, a canonical antioxidant pathway. *In vitro* and *in vivo* experiments confirm that exosomal modulation of this axis elevates GPX4 expression and mitigates joint inflammation and degeneration (189).

Further optimization has been explored through exosome preconditioning. Synovial MSCs pretreated with strontium (Sr) demonstrate enhanced Alix-mediated sorting of miR-143-3p into exosomes, resulting in superior anti-ferroptotic and analgesic effects in temporomandibular joint OA models (190).

Despite these encouraging findings, exosome-based therapies still face technical challenges. These include low production yields, batch-to-batch heterogeneity, and difficulties in purification and cargo standardization (186). Additionally, the dense cartilage ECM and rapid synovial fluid turnover hinder exosome retention and uptake. Strategies such as surface modification, hydrogel encapsulation, or scaffold-assisted delivery may improve targeting and therapeutic persistence in the joint cavity (186).

In summary, MSC- and exosome-based therapies mitigate OA ferroptosis through the METTL3–ACSL4, SNHG7–miR-485-5p–FSP1, and GOT1–CCR2–Nrf2 axes. Addressing bottlenecks in large-scale production, targeted delivery, and *in vivo* stability will be crucial to translating these approaches into clinical practice.

#### 5.1.6 Nanoparticle-based ferroptosis therapies in OA

Nanotechnology has emerged as a promising strategy for enhancing the targeting, efficacy, and bioavailability of ferroptosis-related therapeutics in OA. Several types of nanoplatforms have been designed to deliver small molecules or antioxidants to chondrocytes or inflamed joint tissues.

SM04690 (Adavivint/Lorecivivint), a small molecule currently in Phase III clinical trials, has been formulated as SM04690@ApoF via solvent-free self-assembly into apoferritin nanocages, achieving quantifiable drug loading efficiency and sustained release equilibrium within 12 hours. Through TfR-mediated uptake, SM04690@ApoF targets IL-1β–stimulated chondrocytes, showing a 3.2-fold increase in cellular internalization and 85% retention in the joint at 48 hours post-injection. This formulation enhances ECM metabolism by upregulating Aggrecan and Collagen II while suppressing MMP13 and ADAMTS5. It also inhibits ferroptosis by lowering intracellular iron and ROS levels, increasing GPX4 expression, and reducing ACSL4. In DMM-induced OA models, SM04690@ApoF reduces OARSI scores by 40%, preserves cartilage structure, and promotes proteoglycan deposition. Toxicity studies confirm good biocompatibility, with no cytotoxicity up to 200 μg/mL and no pathological changes in major organs (191). However, the therapeutic efficacy and safety of SM04690 under long-term or repeated dosing remain unverified, and its effects on other joint-resident cells such as synoviocytes and osteoblasts have not been fully explored. The current findings are based on short-term studies in traumatic OA models, limiting their translational generalizability to age-related or metabolic OA. Further studies are needed to assess long-term bio-distribution, sustained joint retention, and broader mechanistic impact across joint tissues.

Other experiments have constructed custom Se- and Ga-based nanoparticles that effectively mitigate cytokine-induced chondrocyte degeneration and ECM breakdown. These nanoparticles exhibit sustained drug release for over 72 hours *in vitro* and achieve 78% joint retention at 48 hours post-injection. In IL-1β-stimulated OA models, they upregulate SOX9 and COMP while suppressing ADAMTS4 activity, and micro-CT analysis shows 35% less subchondral bone loss. They also reduce lipid peroxidation by 52%, restore GPX4 activity to near-normal levels, and preserve mitochondrial ultrastructure. Toxicity evaluations demonstrate an IC50 above 300 μg/mL for joint cells and no adverse effects in 28-day *in vivo* studies. Mechanistically, they activate Nrf2 and block ubiquitin-mediated GPX4 degradation, thereby inhibiting ferroptosis and alleviating cartilage erosion, pain, and gait abnormalities in DMM-induced OA (192). Nevertheless, the precise molecular targets involved in ubiquitination regulation remain unclear, and the role of these nanoparticles in modulating synovial inflammation or subchondral bone remodeling requires further elucidation. Additionally, the long-term fate and metabolic impact of trace elements such as Ga and Se warrant investigation.

A ROS-responsive dextran nanoparticle (FN-CNPs) carrying fenofibrate has demonstrated potent ferroptosis-inhibitory effects in OA. *In vitro*, FN-CNPs reduce lipid peroxidation and ROS levels while restoring GPX4, FSP1, and ACSL3 expression in IL-1β-stimulated chondrocytes. The nanoparticles exhibit a drug loading efficiency of 35.1%, a drug loading content of 3.2%, and an average size of 117.3 ± 1.3 nm, with ROS-triggered release enabling targeted delivery to cartilage tissues. Cytotoxicity assays confirm good biocompatibility up to 200 μg/mL. *In vivo*, intra-articular injection of FN-CNPs in DMM mice slows OA progression and preserves cartilage integrity by modulating ROS levels, antioxidant defense, and lipid metabolism. Histological evaluation of major organs and blood tests reveal no signs of systemic toxicity or inflammatory pathology, supporting a favorable safety profile. These findings highlight FN-CNPs as a promising and well-tolerated nanotherapeutic for OA intervention (193). However, challenges remain in optimizing their cartilage-specific targeting in dense ECM environments, understanding their effects on synovial and subchondral tissues, and validating long-term therapeutic benefits in age-related or metabolic OA models. However, challenges remain in optimizing their cartilage-specific targeting in dense ECM environments, understanding their effects on synovial and subchondral tissues, and validating long-term therapeutic benefits in age-related or metabolic OA models.

### 5.2 Future perspectives and challenges

#### 5.2.1 Ferroptosis-related biomarkers and early diagnosis

In the early stages of OA, disturbances in iron homeostasis and increased lipid peroxidation can already be observed in cartilage and synovium. Developing biomarkers that sensitively detect ferroptosis levels or iron metabolism imbalances could facilitate early OA diagnosis, precise staging, and outcome prediction. Research experiments and bioinformatic analyses suggest that serum or synovial fluid ferritin and GPX4 activity, among others (8, 99), might serve as combined diagnostic indicators, but large-scale cohort studies are essential to confirm their specificity and sensitivity (194).

#### 5.2.2 Crosstalk with other forms of cell death

OA progression involves multiple cell death modalities, including apoptosis, pyroptosis, and necrosis, each of which may intersect or coexist with ferroptosis. Comprehensive therapy must consider not only ferroptosis-specific mechanisms but also other cell death-related pathogenic processes (195). Whether inhibiting pyroptosis or blocking apoptotic pathways exerts synergistic or antagonistic effects on ferroptosis remains unclear. These complex interactions present both opportunities and challenges for OA treatment, necessitating more research on molecular mechanisms and systems biology.

#### 5.2.3 Bottlenecks and opportunities in clinical translation

While ferroptosis-targeted strategies show promise in OA models, the translation to clinical settings remains hindered by key limitations in safety, efficacy, and delivery. For instance, systemic administration of iron chelators such as deferoxamine or deferasirox effectively reduces Fe²^+^ levels and lipid peroxidation but carries the risk of disrupting systemic iron metabolism, potentially causing anemia, immune dysfunction, and impaired wound healing (53, 196, 197). Their poor cartilage specificity and rapid clearance from the joint cavity further limit therapeutic durability.

Similarly, GPX4 activators and indirect inducers such as icariin or vitamin derivatives demonstrate antioxidant and chondroprotective effects in preclinical studies (25, 153, 156). However, GPX4 is a selenoprotein with a unique structural conformation, and no clinically approved small molecule can yet fully restore its catalytic function *in vivo* (23). In addition, exogenous antioxidants often suffer from low bioavailability and off-target distribution, diminishing their efficacy in joint tissues with dense extracellular matrices and limited vascularization (198).

Another challenge lies in modulating key lipid metabolic regulators. Although ACSL4 is consistently upregulated in OA cartilage and linked to ferroptosis progression, its inhibition must be balanced to avoid disrupting normal PUFA metabolism required for membrane integrity and repair (74, 77). LPCAT3, conversely, presents a context-dependent role, acting as either a pro- or anti-ferroptotic modulator depending on the OA stage or inflammatory milieu. This duality complicates its pharmacological targeting. Studies suggest that LPCAT3 suppresses ACSL4 in IL-1β-stimulated chondrocytes, thereby inhibiting ferroptosis and protecting mitochondria, but in other settings, it may exacerbate ferroptotic lipid remodeling (76). Such mechanistic ambiguity necessitates precise stratification of patient populations, possibly through ferroptosis-related biomarkers or transcriptomic profiling.

Altogether, these examples underscore that despite encouraging experimental results, ferroptosis-targeted drugs face multiple translational bottlenecks, including systemic toxicity, insufficient targeting, metabolic compensation, and biological heterogeneity. Overcoming these challenges will require advanced delivery platforms such as nanoparticles or hydrogels, personalized stratification tools such as lipidomic and ncRNA signatures, and rigorous pharmacokinetic validation in long-term OA models.

#### 5.2.4 Novel pharmacotherapies and combination treatment

As a multifactorial degenerative disease, OA often responds poorly to single-target therapies (9). Combination regimens that incorporate iron homeostasis modulation, anti-inflammatory effects, antioxidation, and tissue repair may better address OA’s complicated and dynamic pathogenesis. Novel delivery platforms such as nanocarriers or hydrogels permit targeted co-delivery of multiple agents or genetic material to cartilage and synovium, achieving synergistic treatment while minimizing systemic side effects.

Increasingly, ferroptosis is recognized as central to the multifaceted and dynamic pathophysiology of OA. Therapeutic strategies targeting iron homeostasis, lipid peroxidation, and immunoinflammatory regulation offer promising opportunities for the prevention and treatment of OA. Emerging research on iron chelators, GPX4 activators, TfR1 or FPN targeting, gene therapy, and stem cell-derived exosomes has highlighted their potential to significantly slow cartilage degradation and preserve joint function. However, numerous challenges persist in terms of safety, efficacy, and targeted precision, warranting concerted advances in basic research and clinical trials. As understanding of ferroptosis mechanisms deepens and multidisciplinary innovations progress, a multifaceted approach to targeting ferroptosis may ultimately emerge as a breakthrough for OA management by delaying disease progression and enhancing patients’ quality of life.

#### 5.2.5 Future directions: targeting cell-type specific ferroptosis programs

Emerging single-cell and spatial transcriptomic analyses have revealed that ferroptosis susceptibility varies significantly among different joint-resident cells in OA. For example, a ferroptosis-prone chondrocyte cluster with high expression of ACSL4 and Tf, but low expression of FTH1 and TRPV1, has been identified in severely degenerated cartilage regions (42). This spatial and transcriptional heterogeneity suggests that localized ferroptotic events may drive tissue damage by interfering with cartilage repair and intercellular communication.

We propose that selectively targeting ferroptosis in specific cell populations may enhance therapeutic precision. For instance, upregulating GPX4 in ferroptosis-sensitive chondrocytes, restoring antioxidant defenses in inflammatory synovial macrophages, or rebalancing iron export in subchondral osteoblasts may prevent tissue-specific degeneration. Macrophage-derived exosomes enriched in ferroptosis-inhibitory microRNAs, such as miR-485-5p or miR-214-3p, or exogenously engineered vesicles delivering FSP1, represent promising cell-specific delivery platforms (157, 188, 199, 200).

In addition, novel regulators such as RNA methylation enzymes (METTL3/14) that modulate GPX4 mRNA stability (176), or RhoA-mediated vascular remodeling that enhances iron leakage into subchondral bone (97), may offer new molecular targets. These findings underscore the importance of integrating ferroptosis modulation with joint tissue heterogeneity and the immunometabolic context of OA.

Future research should leverage high-resolution single-cell or spatial omics, functional validation in cell-specific knockout models, and joint-region-targeted delivery systems to develop more personalized and mechanistically guided ferroptosis interventions.

## 6 Conclusion

Finally, we integrate the main points from earlier sections to highlight ferroptosis as a promising research and therapeutic direction in OA (4, 9). OA is one of the primary causes of joint dysfunction and diminished quality of life in the middle-aged and elderly, characterized by a complex, multifactorial pathogenesis (15). Over the past decade, heightened awareness of emerging cell death modes has placed ferroptosis, with its distinctive iron-dependent lipid peroxidation mechanism, at the forefront of OA research (5). This review explores the role of ferroptosis in OA and its potential therapeutic interventions from several perspectives (201).

First, basic insights into OA and ferroptosis confirm that OA progression involves cartilage degeneration, subchondral bone remodeling, and synovial inflammation, in addition to oxidative stress and various cell death modalities. A growing body of evidence reveals pervasive iron homeostasis disruption and elevated lipid peroxidation in the cartilage, subchondral bone, and synovial tissue of OA patients, correlating positively with disease severity and cartilage damage.

Second, the role of metabolic reprogramming in ferroptosis highlights how sustained mechanical stress and inflammation reshape lipid, amino acid, iron, and glucose metabolic pathways in OA chondrocytes and synovial cells. Excess PUFA accumulation, depletion of GSH, and iron overload markedly heighten ferroptosis susceptibility. While metabolic reprogramming may serve as an adaptive response to pathology, it also opens new targets for ferroptosis-directed therapy.

Third, immunometabolic crosstalk in the regulation of ferroptosis highlights the pivotal role of chronic low-grade inflammation in OA pathogenesis. Macrophages and T/B lymphocytes within the synovium, along with their secreted cytokines, interact closely with iron homeostasis and lipid peroxidation pathways. Ferroptosis, in turn, exacerbates inflammation by releasing oxidized byproducts and iron ions, thereby establishing a detrimental feedback loop involving immunity, metabolism, and ferroptosis within the OA microenvironment (105).

Finally, prospects for ferroptosis-based interventions and future directions point to several promising treatment avenues, including regulating iron homeostasis via chelators or TfR1/FPN modulation, using antioxidants and lipid peroxidation inhibitors to target GPX4 and ACSL4, or employing gene therapies and stem cell/exosome approaches to combat ferroptosis. Moreover, properly harnessing the Nrf2/HO-1 axis while managing its potential pro-ferroptotic risks may help maintain redox balance and curb iron overload, thereby offering an additional strategy to alleviate ferroptosis-driven damage in OA (202). Nonetheless, numerous challenges remain. These include the development of sensitive and specific ferroptosis-related biomarkers and imaging techniques for early OA detection; a deeper understanding of the interplay between ferroptosis and other cell death pathways such as apoptosis, pyroptosis, and necrosis; comprehensive safety evaluations and the design of personalized therapeutic regimens for clinical application; and the advancement of innovative drug delivery systems, such as nanocarriers and hydrogels, that integrate strategies for regulating iron homeostasis, reducing inflammation, counteracting oxidative stress, and promoting cartilage regeneration.

Collectively, ongoing research on OA and ferroptosis is advancing rapidly, offering a novel perspective on the complex interplay among cartilage degradation, synovial inflammation, and subchondral bone remodeling. A more comprehensive understanding of the molecular networks governing ferroptosis in OA will facilitate the development of refined therapeutic strategies at multiple levels, potentially leading to improved structural and functional outcomes for patients. As mechanistic insights into iron homeostasis, lipid peroxidation, and immunometabolic interactions continue to deepen, alongside progress in drug delivery, gene editing, and regenerative medicine, therapies targeting ferroptosis may represent a promising new paradigm in OA management, ultimately enhancing joint function and overall quality of life for affected individuals.
